# Patient-Specific Computational Modeling of Upper Extremity Arteriovenous Fistula Creation: Its Feasibility to Support Clinical Decision-Making

**DOI:** 10.1371/journal.pone.0034491

**Published:** 2012-04-04

**Authors:** Aron S. Bode, Wouter Huberts, E. Marielle H. Bosboom, Wilco Kroon, Wim P. M. van der Linden, R. Nils Planken, Frans N. van de Vosse, Jan H. M. Tordoir

**Affiliations:** 1 Department of Surgery, Maastricht University Medical Center, Maastricht, The Netherlands; 2 Department of Biomedical Engineering, Eindhoven University of Technology, Eindhoven, The Netherlands; 3 Department of Biomedical Engineering, Maastricht University Medical Center, Maastricht, The Netherlands; 4 Philips Research Eindhoven, Healthcare Information Management Group, Eindhoven, The Netherlands; 5 Academic Medical Center Amsterdam, Department of Radiology, Amsterdam, The Netherlands; University of Sao Paulo Medical School, Brazil

## Abstract

**Introduction:**

Inadequate flow enhancement on the one hand, and excessive flow enhancement on the other hand, remain frequent complications of arteriovenous fistula (AVF) creation, and hamper hemodialysis therapy in patients with end-stage renal disease. In an effort to reduce these, a patient-specific computational model, capable of predicting postoperative flow, has been developed. The purpose of this study was to determine the accuracy of the patient-specific model and to investigate its feasibility to support decision-making in AVF surgery.

**Methods:**

Patient-specific pulse wave propagation models were created for 25 patients awaiting AVF creation. Model input parameters were obtained from clinical measurements and literature. For every patient, a radiocephalic AVF, a brachiocephalic AVF, and a brachiobasilic AVF configuration were simulated and analyzed for their postoperative flow. The most distal configuration with a predicted flow between 400 and 1500 ml/min was considered the preferred location for AVF surgery. The suggestion of the model was compared to the choice of an experienced vascular surgeon. Furthermore, predicted flows were compared to measured postoperative flows.

**Results:**

Taken into account the confidence interval (25^th^ and 75^th^ percentile interval), overlap between predicted and measured postoperative flows was observed in 70% of the patients. Differentiation between upper and lower arm configuration was similar in 76% of the patients, whereas discrimination between two upper arm AVF configurations was more difficult. In 3 patients the surgeon created an upper arm AVF, while model based predictions allowed for lower arm AVF creation, thereby preserving proximal vessels. In one patient early thrombosis in a radiocephalic AVF was observed which might have been indicated by the low predicted postoperative flow.

**Conclusions:**

Postoperative flow can be predicted relatively accurately for multiple AVF configurations by using computational modeling. This model may therefore be considered a valuable additional tool in the preoperative work-up of patients awaiting AVF creation.

## Introduction

Patients suffering from end-stage renal disease (ESRD) depending on hemodialysis (HD) therapy require a functional vascular access (VA) [1], which can be provided by creation of an arteriovenous fistula (AVF), creation of an arteriovenous graft (AVG), or the insertion of a central venous catheter (CVC). Since the use of prosthetic graft material (AVG and CVC) is associated with reduced patency rates and higher mortality rates [2], [3], guidelines advocate the use of native vessels for VA creation [4]. As a result, the preferred option for VA creation consists of surgically connecting an artery with a vein in either the lower arm (e.g. by connecting the radial artery with the cephalic vein) or the upper arm (e.g. by connecting the brachial artery with either the cephalic or basilic vein at the level of the elbow), with a sequential order of preference of 1) the radiocephalic fistula [RC-AVF], 2) the brachiocephalic fistula [BC-AVF], and 3) the brachiobasilic fistula [BB-AVF] [5].

An important downside of AVF creation is the significant probability of early thrombosis or nonmaturation (20–50%) due to insufficient flow enhancement, particularly in lower arm AVF's [6], [7], and excessive postoperative flow enhancement resulting in steal syndrome and cardiac failure (up to 20%) in elbow AVF's [8], [9]. In an effort to limit these complications, an extensive preoperative duplex ultrasound (DUS) evaluation of the upper extremity vascular tree is performed to select the most suitable site for AVF creation [10]. Unfortunately, flow related complications persist and additional interventions are often needed to make the AVF suitable for HD treatment [11].

Following AVF surgery, flow enhancement is determined by multiple factors and thus differs between patients. Geometrical factors (e.g. vascular diameters and lengths), vessel topology (e.g. number and caliber of venous sidebranches), the resistance of the capillary beds (peripheral resistances), and structures such as stenoses and the anastomosis, all influence the resistance to blood flow, and are therefore believed to be accountable for the observed flow increase. Hence, the currently performed discrete diameter measurements of upper extremity vasculature only partially represent the hemodynamic resistances that influence flow enhancement.

Computational modeling allows to investigate patient-specific hemodynamics by employing physical laws for quantitative integration of the multiple prognostic factors, and has already proved to be of assistance in aortic aneurysmal disease [12], [13], in cerebral disease [14], [15], and coronary artery disease [16], [17]. Although models have been used previously to gain insight in VA hemodynamics and pathologies, or disease progression associated with it [18], [19], [20], [21], predictive models, aiming for a more accurate risk-estimation regarding the development of flow related complications, have not been used.

Previously, within the 7^th^ Framework Program of the European Commission, a pulse wave propagation model has been developed that is able to simulate pressure and flow changes after AVF creation that are also observed in clinical setting [22]. This pulse wave propagation model has been validated against a silicone model of the aorta, arm arteries and veins in which AVF procedures were mimicked [23]. It has been shown that the main features of experimental flow and pressure waveforms, both before and after AVF creation, were adequately simulated. Subsequently, this model was adapted to patient-specific conditions for 10 patients suffering from ESRD to show its potential to support clinical decision-making [22]. It was shown that the model was able to select the same AVF configuration as an experienced surgeon in 9/10 patients. However, predicted postoperative flows differed in 4/10 patients when compared to Doppler ultrasound flow measurements directly after surgery. These differences might be attributed to 1) neglecting vascular adaptation and autoregulation of the capillary beds, 2) uncertainties in the postoperative flow measurements, or 3) uncertainties in model input parameters which might results in uncertainties in output. These latter uncertainties in the model predictions were not considered previously. The purpose of this study was to determine if the pulse wave propagation model is able to predict the immediate postoperative flow, and to examine its feasibility to support decision-making while considering the uncertainties of the flow predictions due to input parameter uncertainties.

## Methods

### Study population

Twenty-five consecutive patients suffering from ESRD awaiting their first VA creation were enrolled in this prospective observational study. The study was approved by the medical ethical committee of the Maastricht University Medical Center, and written informed consent was obtained from all individuals prior to enrolment in the study. All clinical investigations have been performed according to the principles expressed in the Declaration of Helsinki.

### Pulse wave propagation model

**Figure 1:**
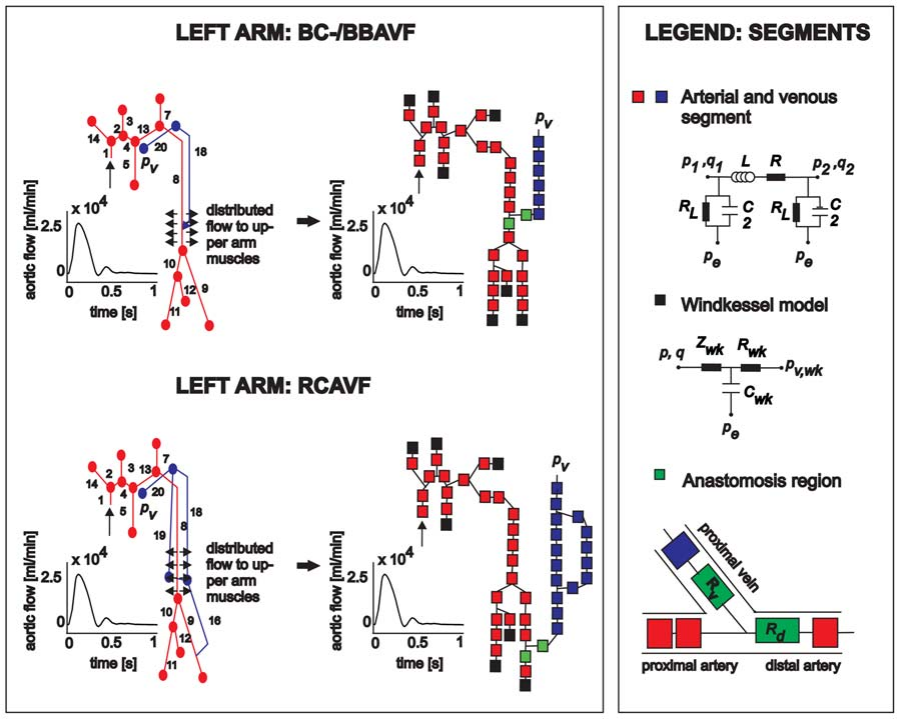
Left arm vasculature divided into arterial, venous and anastomosis segments (middle). These segments locally describe the relation between pressure *p* and flow *q* via a lumped parameter approach (right), and consists of a resistor *R* (viscous resistance to blood flow), a resistor *R_L_* (viscous resistance of blood flow to small side-branches), an inductor L (blood inertia) and a capacitor *C* (vascular compliance). The anastomosis is modeled with two nonlinear resistors *R_v_* and *R_d_*. The windkessels consist of two resistors, *Z_wk_ and R_wk_* (together the peripheral resistance) and a capacitor C_wk_ (peripheral compliance). This figure is adapted from Huberts et al.

The pulse wave propagation model used here, has been described in detail in previous work [22]. In short, the model simulates pressure and flow waveforms on several arterial and venous locations of the upper extremity. Depending on the site (left or right) and the AVF configuration (radiocephalic AVF [RC-AVF], brachiocephalic AVF [BC-AVF], brachiobasilic AVF [BB-AVF]), inflow arteries and outflow veins of interest were included in the computational domain (Table 1, Figure 1). Each vessel of the computational domain was divided into segments with a maximum length of 5 cm, describing the local relation between pressure and flow via a lumped parameter approach. Such a lumped segment consists of a resistor R, representing the viscous resistance to blood flow through the vessel segment, a resistor R_L_, representing the resistance to blood flow through small sidebranches not modeled in detail, an inductor L, representing the inertia of the blood and a capacitor C, representing the vascular compliance (i.e. storage capacity of the vessel). For the anastomosis, a segment was used consisting of nonlinear resistors that depend on anastomosis angle and blood flow. Arteries not included in the computational domain as well as the peripheral vascular beds were modeled by windkessel elements with a specific resistance and compliance. As boundary conditions, an intravenous pressure was prescribed at the subclavian vein, whereas postoperative inflow was prescribed at the aorta. Since the latter is preoperatively unknown, the aortic flow was measured preoperatively and iteratively updated by scaling the preoperative waveform until the postoperative mean aortic pressure was restored to the preoperative level (baroreflex). All other preoperative model parameters (e.g. peripheral resistances) were kept constant.

**Table 1:** The names of all vessels included in the computational domains.

Number [-]	Vessel name [-]	Number [-]	Vessel name [-]
**1**	Ascending aorta	11	Distal Ulnar artery
**2**	Aortic arch A1	12	Interosseus artery
**3**	Left Carotid artery	13	Left Subclavian artery
**4**	Aortic arch A2	14	Innominate artery
**5**	Thoracic aorta	15	Right carotid artery
**6**	Right subclavian artery	16	Distal cephalic vein
**7**	Vertebral artery	17	Median Cubital vein
**8**	Axillary and Brachial artery	18	Proximal Cephalic vein
**9**	Radial artery	19	Basilic vein
**10**	Proximal Ulnar artery	20	Axillary and Subclavian vein

### Personalization of the pulse wave propagation model

To personalize the input parameters of the pulse wave propagation model, patient-specific anatomy (vessel length, vessel diameters, vessel wall thickness) and mechanical characteristics of the vessels (vascular compliance) were mandatory. Furthermore, information on anastomosis configuration (location, angle), windkessel parameters, blood properties (density and dynamic viscosity), intravenous pressure and preoperative aortic flow waveform were required. However in clinical practice, it is impossible to assess all these parameters for every patient. Fortunately, not all parameters are equally important for the prediction of postoperative flow enhancement; model parameters that need to be measured patient-specifically opposed to model parameters that can be estimated from literature, were identified previously in a sensitivity analysis [24].

**Figure 2:**
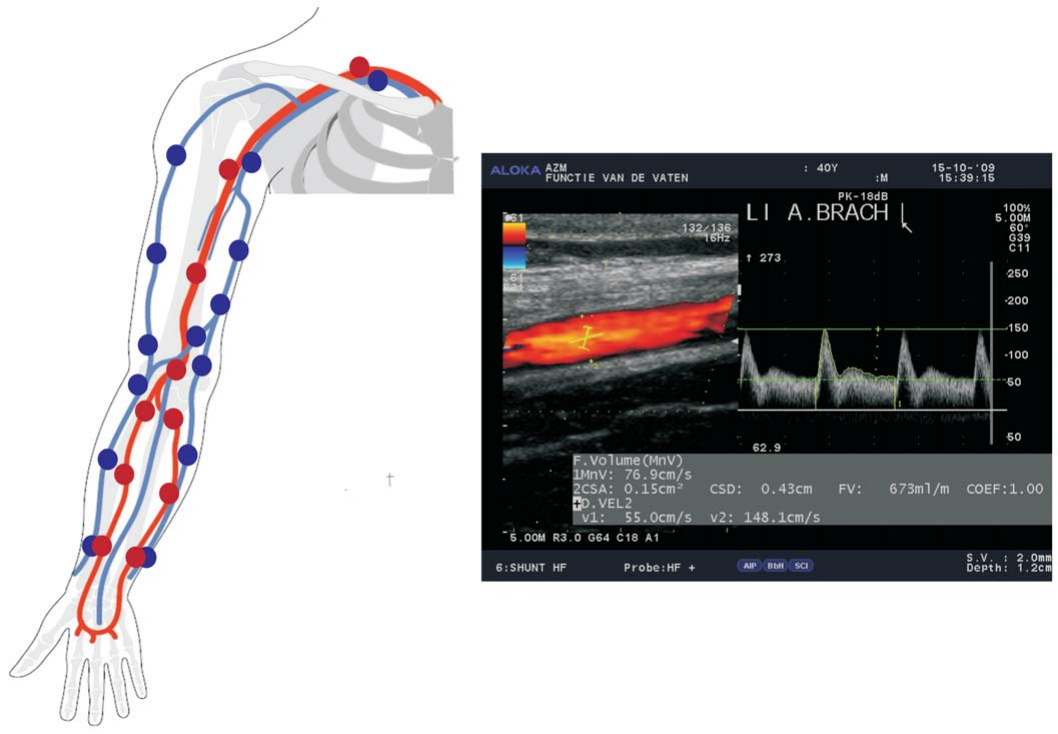
Schematic picture of the locations of the diameter measurements in the preoperative DUS examination (left). At the right a schematic picture of the postoperative flow determination is presented.

Model parameters were, by considering the insights obtained from the sensitivity analysis, chosen as follows. Arterial lengths were based on a generic geometry taken from Stergiopulos et al [25]. Venous lengths are considered equal to arterial lengths on the same anatomical location. Upper extremity vascular diameter measurements were obtained patient-specifically on discrete locations by performing an extensive preoperative duplex ultrasound examination which was already part of clinical routine in our hospital. The diameter measurement locations are schematically shown in Figure 2. The diameters of the veins in the upper extremity are measured by using a tourniquet to increase the reproducibility of the measurements [26]. Arteries and veins exceeding a 2 mm threshold are considered vessels with a proper caliber for AVF creation. For details about the duplex examination, we refer to Bode et al [27]. Missing diameters of the arm vasculature were obtained by linear inter-, or extrapolation. Diameters of the aorta and its primary branches were based on literature and scaled according to upper extremity arterial diameters [25]. Vessel wall thicknesses were derived from wall thickness-to-radius ratios: a ratio of 15% was used for the subclavian, axillary and brachial artery, whereas a ratio of 20% was used for the radial, ulnar and interosseus artery [28], [29], [30]. The ratios of all other arteries were based on literature [25]. For veins a ratio of 10% was chosen [31].

Mechanical properties of the upper extremity vessels were characterized by vascular compliance. For this, in addition to wall thickness and diameter, the Young's modulus was required [22], [32]. The Young's modulus of the brachial artery was determined via arterial distensibility, which was assessed by a Picus ultrasound machine equipped with ARTLAB software (ESAOTE, Maastricht, The Netherlands). For each patient, vessel wall distension over the cardiac cycle was measured using a wall-tracking technique in combination with continuous, non-invasive pressure registration (Nexfin, BMEye, Amsterdam, The Netherlands). The Young's modulus of the brachial artery was applied for the compliance of all arterial arm segments, whereas for the aorta and veins Young's moduli were based on literature [22], [25].

The location of the anastomosis was set to 5 cm proximal to the wrist in case of a lower arm AVF and 5 cm proximal to the elbow bifurcation in case of an upper arm AVF, which are conventional locations for AVF surgery. The angle of the AVF between the proximal artery and vein cannot be influenced by the surgeon due to anatomical restrictions. For simulations this angle was set to 45 degrees, because unpublished data of postoperative MR images show that a typical angle varies between 30 and 60 degrees. Windkessel parameters were personalized by using mean arterial pressure and mean arterial flows in the aorta, brachial, radial, and ulnar artery. Mean flows were obtained by preoperative MR flow measurements (Philips Healthcare, Best, The Netherlands), whereas mean arterial pressure was assessed by using the Nexfin.

Blood was considered to behave as an incompressible Newtonian fluid with a density of 1×10^3^ kg/m^3^ and a dynamic viscosity of 3×10^−3^ Pa·s. Intravenous pressure at the subclavian vein was set to 10 mmHg [33].

In Table 2 an overview of all the measurements performed on each patient and their required examination time are presented. The measurements consist of clinical routine advocated by guidelines and additional measurements performed in the context of this study.

**Table 2:** Patient-specific measurements performed in the context of the study.

	Examination	Duration
**Current clinical routine**	Vessel mapping with DUS	60 min
	Postoperative flow measurements with DUS	10 min
**Additional measurements**	Fingerpressure and distensibility measurements	15 min
	Preoperative MR flow measurements	20 min

### Analysis

#### Absolute postoperative flow prediction

In order to quantitatively determine the accuracy of flow predictions, predicted flows of the created AVF configuration were compared with observed postoperative flows as measured with DUS one week after surgery. In this perspective, the uncertainty of the flow prediction resulting from input parameter uncertainty is evaluated by means of Monte Carlo simulations as described by Huberts et al [24], and expressed through the 25^th^–75^th^ percentile interval; the large number of Monte Carlo runs made it difficult to quantitatively determine normality. The uncertainties applied to the model input parameters are shown in Appendix S1. For each Monte Carlo simulation, a value is assigned to each model input parameter by sampling the uncertainty domains (initial value ± the applied uncertainty) by means of Latin Hypercube sampling so that a full coverage of model parameter input space is guaranteed. The actual postoperative brachial artery flow was assessed by multiplying the time-averaged outer envelope of the Doppler velocity spectrum with the local cross-sectional area, which is obtained from a B-mode image (Figure 2). The velocity profile throughout the vessel lumen is not exactly known but should lie between a flat and a parabolic velocity profile. To correct for the velocity profile, the measured brachial artery flow is presented in this study between 0.5 (parabolic) and 1 (flat) times the assessed flow.

### Arteriovenous fistula configuration

To examine the model's feasibility to support decision-making , three different AVF configurations (RC-AVF, BC-AVF, BB-AVF) were considered for each patient, and evaluated with respect to their postoperative flow directly after surgery. In lower arm fistulas the immediate postoperative flow is approximately 60–70% of the flow after successful maturation, whereas this is 90–100% in elbow fistulas [34]. Furthermore, postoperative flows larger than 30% of the cardiac output are associated with an increased risk for cardiac failure and hand ischemia [8], [9]. As a result, AVF configurations resulting in a predicted postoperative flow between 400 and 1500 ml/min were considered by the model as an option for AVF creation. When more AVF configurations resulted in a flow exceeding 400 ml/min, the sequential order of preference was RC-AVF, BC-AVF, and BB-AVF. To objectivate the model's capability to identify the optimal location for AVF creation, the suggested AVF configuration was compared with the choice of a surgeon with ample experience in VA surgery (more than 1000 AVF creations).

## Results

### Absolute postoperative flow prediction

Postoperative flow predictions could be compared to clinically measured flows in 23 of 25 patients. One patient (#15) was excluded from this analysis because graft material was used for creation of the VA conduit, which is not supported by the computational model. A second patient (#21) was excluded because of immediate thrombosis and, as a result, no postoperative flow measurement was available.

**Figure 3:**
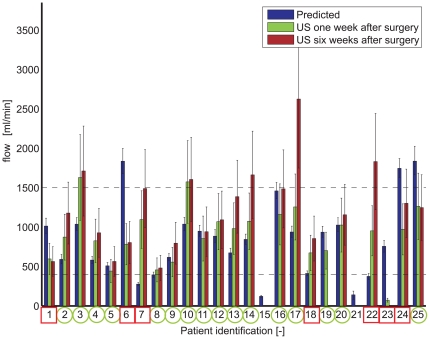
Schematic visualization of predicted and measured postoperative flows for the AVF configuration created by the vascular surgeon. The error bars in predicted flow are the result of inaccuracies in input parameters, while the error bars in postoperative flow are the result of measurement inaccuracies^21^. For patient #15 prosthetic graft material was used for VA creation. For patient #21 no postoperative flow measurements could be obtained due to immediate thrombosis. A green circle around the patient identification represents overlap between predicted and measured postoperative flow (16 patients). A red square around the patient identification represents a discrepancy between predicted and measured postoperative flow (7 patients). Note that patient #24 received an alternative AVF configuration (cephalic vein was anastomosed with the ulnar artery on the upper arm due to a high brachial artery bifurcation).

Figure 3 shows predicted flows versus measured flows one week after surgery. In addition, the flow measurement at six weeks is visualized to gain insight into flow enhancement during the maturation phase. At one week, predicted and measured flow show overlap in 16 patients (16/23: 70%). In patient #1, #6, #23, and #24 the predicted flow is an overestimation of the measured flow, while in patient #7, #18, and #22 the predicted flow is an underestimation of the measured flow. In patient #1 a significant hematoma was identified during the immediate postoperative duplex control, whereas in patient #23 postoperative thrombosis was observed one week after surgery.

### Arteriovenous fistula configuration

In 4 of 25 patients postoperative brachial artery flow could not be simulated for all three AVF configurations because the cephalic vein could not be visualized preoperatively due to pre-existing thrombosis (patient #19, #23, and #25) or because the computations did not converge for all Monte Carlo simulations (patient #24). As in these patients not all AVF configurations could be simulated by the model, they were excluded on beforehand regarding the AVF *configuration* analysis.

**Figure 4:**
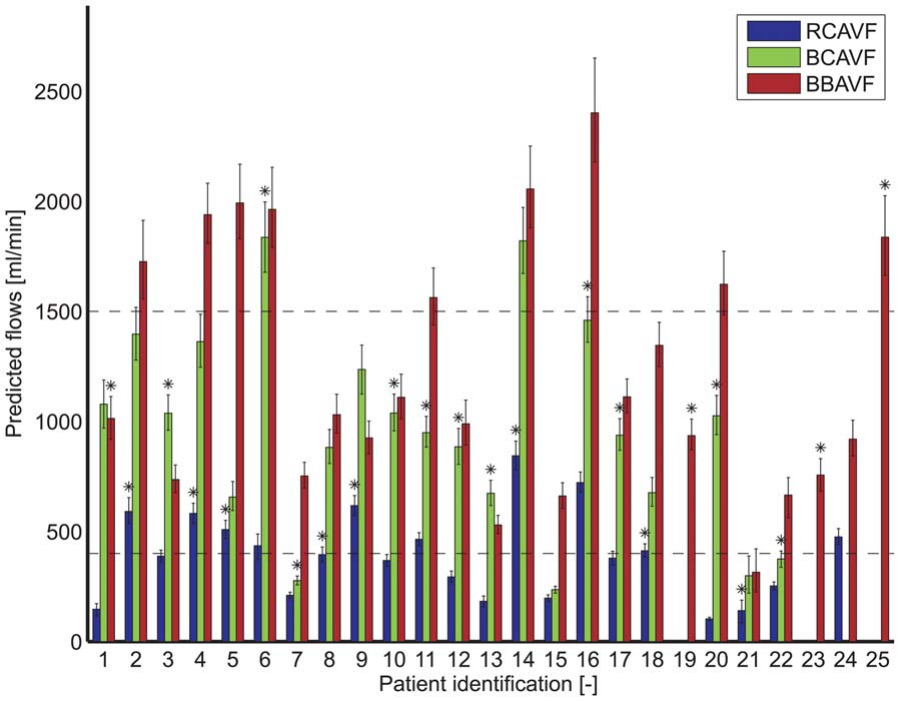
The predicted postoperative flows for a RCAVF, BCAVF and BBAVF configuration. The flows are presented as the median of all Monte Carlo simulations with their 25^th^ and 75^th^ percentile interval. In 4 patients postoperative brachial artery flow could not be simulated for all three AVF configurations because essential patient-specific data were missing due to thrombosis of the cephalic vein (patient #19, #23, and #25) or because the computations did not converge for all Monte Carlo simulations (patient #24). An asterix represents the fistula configuration chosen by the surgeon.

In the remainder of patients (N = 21), the model suggested an upper arm or lower arm AVF configuration in agreement with the choice of the surgeon for 16 patients (16/21: 76%). In five patients the suggestion of the model and the choice of the surgeon were different (Figure 4); in patients #6, #11, and #16 model predictions may have allowed for a lower arm AVF, while the surgeon created upper arm AVF's. Conversely, in patient #8 the surgeon created a lower arm AVF, while the model suggests to create an upper arm AVF. Also in patient #21 a lower arm AVF was created, whereas the model predicts a too low postoperative flow for all configurations. These low flow predictions might have been indicative for the early failure as observed in patient #21.

When differentiating between RC-AVF, BC-AVF, and BB-AVF, the model suggests the same AVF configuration as the surgeon in 12 patients (12/20: 60%) (Figure 4). For this analysis, one additional patient (#15) was excluded, because during the surgical procedure immediate thrombosis occurred for the intended BB-AVF configuration and prosthetic graft material was used to create the VA. In addition to the five previously mentioned patients, there is a discrepancy between the suggested configuration of the model and the choice of the surgeon with respect to a BC-AVF or BB-AVF configuration in three patients (#1, #7, and #22).

## Discussion

In this study we investigated the accuracy and feasibility of a pulse wave propagation model to support decision-making in AVF surgery by predicting immediate postoperative brachial artery flow for multiple AVF configurations, , while considering the uncertainty in the flow predictions resulting from uncertainties in the model input parameters. By using the described model, postoperative brachial artery flow could be estimated in 70% of all patients without subjecting the patient to excessive additional preoperative measurements.

In clinical routine, preoperative mapping of upper extremity vasculature with DUS is considered the method of choice to identify the most suitable site for VA creation, and its clinical implementation has been associated with a significant reduction of postoperative failure rates [35], [36]. Nevertheless, complications related to either insufficient flow enhancement, particularly in lower arm fistulas, and to excessive flow enhancement, mainly in upper arm fistulas, remain responsible for AVF failure in a significant number of patients [6], [7], [8], [9]. Therefore, alternative modalities to decrease the incidence of flow related complications have become of interest.

Pulse wave propagation models have been shown in both experimental setup and in vivo to be able to simulate pressure and flow waveforms on multiple locations [37], [38]. Some studies have reported the use of pulse wave propagation models for prediction of outcome after vascular surgery [39], [40]. However, the application of predictive models is still in its infancy. Regarding AVF surgery, prior work of our group focused on the development and experimental validation of a pulse wave propagation model in which pressure and flow distributions in the upper extremity vasculature can be simulated [22]. This way, hemodynamic consequences of AVF creation can be evaluated by taking multiple prognostic factors, as well as their complex interplay into consideration, instead of focusing on discrete diameter measurements. As already indicated by a previous pilot study, the pulse wave propagation model showed potential to suggest the most suitable AVF configuration by patient-specifically predicting postoperative flow [22]. In this study, postoperative flow predictions of the model were subject of clinical validation while considering the uncertainty of the model predictions due to input parameter uncertainties.

By using the pulse wave propagation model, overlap between predicted and observed postoperative flow was observed in 70% of the patients. Predicted flows in 4 patients overestimate the flows measured with ultrasound at one week, while the predicted flows of 3 patients underestimate the measured flows. Overestimation of flow in patient #1 and #23 might be explained by the occurrence of a postoperative hematoma compressing the venous outflow trajectory, and by unexpected early thrombosis, respectively. Other possible explanations for overestimation of postoperative flow might be stenotic segments, curvature or kinking not detected with routine DUS examination. These vessels abnormalities are not included in the computational model but would increase the resistance to blood flow in the VA conduit. An improved depiction of such structures might further improve absolute flow predictions. A possible solution might be to perform a MRA of the upper extremity vasculature which allows for assessment of vascular diameter over the complete vascular trajectory, and for the identification of stenoses, curvature and kinking [41], [42]. In addition, MRA might be beneficial in patients in whom vascular anatomy has been influenced by previous VA creation. The model can easily be adapted to deal with resulting extra pressure drops and altered vascular geometries. Underestimation of postoperative flow might be caused by the lack of vascular adaptation laws in the computational model. In patient #7 and #22 , this might be indicated by the large flow enhancement from week one to week six. In addition, flow mediated dilatation and autoregulation are not incorporated in the model. Although model improvements may result in a better description of postoperative hemodynamics, they also require more (advanced) patient-specific measurements to acquire all input parameters, and increase the burden on the patient.

According to the observations in the current study, the suggestion of the patient-specific wave propagation model for an upper or lower arm AVF configuration already corresponds to the selection of an experienced surgeon in 76% of the patients. In three patients the surgeon decided to create an upper arm AVF, whereas the model allowed for creation of a lower arm AVF. When this additional information would have been available to the surgeon at the time of VA planning, the surgeon might have considered to preserve proximal vessels for future VA procedures by primary creation of a lower arm AVF. Conversely, upper arm AVF creation was suggested in two patients while a lower arm AVF procedure was performed. In one of these patients, immediate thrombosis was observed, while in the other patient the measured flow was slightly above the threshold of 400 ml/min, as was predicted by the model. This additional information might also have changed the surgeon's choice when known in advance. In this perspective, computational modeling might be considered a potential valuable tool in the preoperative work-up, in addition to the currently performed diameter measurements, especially for surgeons that are less experienced in creating AVF's. However, a prospective randomized clinical trial is required to establish the additional value of the model in routine clinical practice.

Considering the differentiation between BC-AVF and BB-AVF, a discrepancy between model and surgeon was observed in three patients: two BC-AVF's were created where the model suggested a BB-AVF configuration, and one BB-AVF was created while a BC-AVF appeared to be feasible according to the model. Although the model might already be able to differentiate between an upper and lower arm AVF, it appears to be more difficult to distinguish between two upper arm AVF configurations. This might be caused by the fact that in the computational model the inflow and outflow trajectories are geometrically similar except for venous diameters. To improve the differentiation between these upper arm AVF's, more accurate venous diameter measurements are required. Moreover, incorporation of accessory veins, the deep venous system and local vascular adaptation might further improve the differentiation.

The study presented here has limitations. Firstly, the number of enrolled patients is not sufficient to determine the predictive value, sensitivity and specificity of the model as an additional tool in the preoperative work-up in patients awaiting AVF creation. For this, a large prospective randomized clinical trial needs to be initiated. However for the aims of this study, i.e. comparing the patient-specific predictions with measurements and assessing the feasibility of the model as potential valuable tool, the number of enrolled patients suffices. In this study, it was more important to determine the uncertainty in the predictions due to uncertainties in the input. In addition, to ensure that conclusions hold for all AVF patients, it was important to cover a whole range of postoperative flows and AVF configurations which are representative for the possible flow regimes after AVF surgery. Both were properly assessed in this study. As a further limitation, one might consider the difficulty of model personalization, since in clinical practice not all input parameters can be obtained for each patient with sufficient accuracy (e.g. windkessels). The reason for this is the limited availability of measurement modalities, and moreover, because the additional burden on the patient should be minimized. Fortunately, the previous sensitivity analysis showed that some input parameters are more important than others [24]. As a result, half of all input parameters could be derived from literature. To this end assumptions had to be made, for which additional sensitivity analysis showed that these do not significantly alter outcome. Another limitation is that the model in this study predicts the immediate postoperative flow as indicator for successful maturation, possible hand ischemia and/or cardiac failure. In this perspective, the model can already be useful to support decision-making in AVF surgery. However, to better predict these long-term effects, long-term adaptation processes as described by e.g. Roy-Chaudhury et al. [43] should be incorporated in the model. Unfortunately, the process of maturation and cardiac adaptation is not fully understood yet. Furthermore, increasing the complexity of the model will not necessarily result in better flow predictions because more patient-specific model input parameters are required, which are all hampered by uncertainty. Finally, the comparison between simulations and measurements is hampered by the uncertainty in the flow measurements. In this study, the postoperative flow measurements were presented with a correction for the velocity profile but the uncertainty in the cross-sectional area used in the calculation was neglected. To strengthen comparison in future studies, it might be worthwhile to improve the DUS flow measurements.

In conclusion, we have demonstrated that a patient-specific pulse wave propagation model can be considered potentially beneficial in the preoperative work-up of patients awaiting VA creation, since postoperative flow can be predicted relatively accurately for multiple AVF configurations. Future effort should focus on acquiring a more detailed overview of patient-specific vasculature to capture vascular pathology and geometry, and for simulation of the maturation process, adaptation laws should be incorporated. To establish the additional value of modeling in clinical decision-making, a large prospective randomized clinical trial needs to be performed.

## Supporting Information

Appendix S1 **Monte Carlo simulations (see Robert et al.1 for details about Monte Carlo simulations) are used to determine the uncertainty in model predictions resulting from uncertainty in the input parameters assessed by measurements or from literature by applying certain assumptions.** The uncertainties in the model parameters and their motivation are shown.
(DOC)

## References

[1] Sidawy AN, Gray R, Besarab A, Henry M, Ascher E (2002). Recommended standards for reports dealing with arteriovenous hemodialysis accesses.. J Vasc Surg.

[2] Tordoir JHM, Canaud B, Haage P, Konner K, Basci A (2007). EBPG on Vascular Access.. Nephrol Dial Transplant.

[3] Pisoni RL, Arrington CJ, Albert JM, Ethier J, Kimata N (2009). Facility hemodialysis vascular access use and mortality in countries participating in DOPPS: an instrumental variable analysis.. Am J Kidney Dis.

[4] National Kidney Foundation Kidney Disease Outcomes Quality Initiative: clinical practice guidelines for vascular access, update 2006.. Am J Kidney Dis.

[5] Bode A, Tordoir J, Azar A (2012). Vascular Access for Hemodialysis Therapy.. Modeling and Control of Dialysis Systems.

[6] Allon M, Robbin ML (2002). Increasing arteriovenous fistulas in hemodialysis patients: problems and solutions.. Kidney Int.

[7] Tordoir JHM, Rooyens P, Dammers R, van der Sande FM, de Haan M (2003). Prospective evaluation of failure modes in autogenous radiocephalic wrist access for haemodialysis.. Nephrol Dial Transplant.

[8] Tordoir JH, Dammers R, van der Sande FM (2004). Upper extremity ischemia and hemodialysis vascular access.. Eur J Vasc Endovasc Surg.

[9] Wijnen E, Keuter XH, Planken NR, van der Sande FM, Tordoir JH (2005). The relation between vascular access flow and different types of vascular access with systemic hemodynamics in hemodialysis patients.. Artif Organs.

[10] Malovrh M (2002). Native arteriovenous fistula: preoperative evaluation.. Am J Kidney Dis.

[11] Lee T, Ullah A, Allon M, Succop P, El-Khatib M Decreased Cumulative Access Survival in Arteriovenous Fistulas Requiring Interventions to Promote Maturation.. Clin J Am Soc Nephrol.

[12] Vorp DA (2007). Biomechanics of abdominal aortic aneurysm.. J Biomech.

[13] Breeuwer M, de Putter S, Kose U, Speelman L, Visser K (2008). Towards patient-specific risk assessment of abdominal aortic aneurysm.. Med Biol Eng Comput.

[14] David T, Moore S (2008). Modeling perfusion in the cerebral vasculature.. Med Eng Phys.

[15] Cebral JR, Castro MA, Burgess JE, Pergolizzi RS, Sheridan MJ (2005). Characterization of cerebral aneurysms for assessing risk of rupture by using patient-specific computational hemodynamics models.. AJNR Am J Neuroradiol.

[16] Hunter PJ, Pullan AJ, Smaill BH (2003). Modeling total heart function.. Annu Rev Biomed Eng.

[17] Gijsen FJ, Wentzel JJ, Thury A, Lamers B, Schuurbiers JC (2007). A new imaging technique to study 3-D plaque and shear stress distribution in human coronary artery bifurcations in vivo.. J Biomech.

[18] Krueger U, Zanow J, Scholz H (2000). Comparison of Two Different Arteriovenous Anastomotic Forms By Numerical 3D Simulation of Blood Flow.. Int J Angiol.

[19] Krueger U, Zanow J, Scholz H (2002). Computational fluid dynamics and vascular access.. Artif Organs.

[20] Zanow J, Petzold K, Petzold M, Krueger U, Scholz H (2006). Flow reduction in high-flow arteriovenous access using intraoperative flow monitoring.. J Vasc Surg.

[21] Zanow J, Krueger U, Reddemann P, Scholz H (2008). Experimental study of hemodynamics in procedures to treat access-related ischemia.. J Vasc Surg.

[22] Huberts W, Bode AS, Kroon JW, Planken RN, Tordoir JHM (2011). A pulse wave propagation model to support decision-making in vascular access planning in the clinic.. Med Eng Phys.

[23] Huberts W, Van Canneyt K, Eloot S, Segers P, Verdonck P (2011). Experimental validation of a pulse wave propagation model to predict hemodynamics after vascular access surgery.. J Biomech.

[24] Huberts W, de Jonge C, van der Linden WPM, Inda MA, Passera K (2011). A sensitivity study to reduce the number of input parameters in a personalized pulse wave propagation model for AVF surgery planning..

[25] Stergiopulos N, Young DF, Rogge TR (1992). Computer simulation of arterial flow with applications to arterial and aortic stenoses.. J Biomech.

[26] Planken RN, Keuter XH, Kessels AG, Hoeks AP, Leiner T (2006). Forearm cephalic vein cross-sectional area changes at incremental congestion pressures: towards a standardized and reproducible vein mapping protocol.. J Vasc Surg.

[27] Bode A, Caroli A, Huberts W, Planken N, Antiga L Clinical study protocol for the arch project Computational modeling for improvement of outcome after vascular access creation.. J Vasc Access.

[28] Kaiser DR, Mullen K, Bank AJ (2001). Brachial artery elastic mechanics in patients with heart failure.. Hypertension.

[29] Ku YM, Kim YO, Kim JI, Choi YJ, Yoon SA (2006). Ultrasonographic measurement of intima-media thickness of radial artery in pre-dialysis uraemic patients: comparison with histological examination.. Nephrol Dial Transplant.

[30] Mourad JJ, Girerd X, Boutouyrie P, Laurent S, Safar M (1997). Increased stiffness of radial artery wall material in end-stage renal disease.. Hypertension.

[31] McArdle WD, Katch FI, Katch VL (1996). Exercise physiology; energy, nutrition, and human performance.

[32] Westerhof N, Bosman F, De Vries CJ, Noordergraaf A (1969). Analog studies of the human systemic arterial tree.. J Biomech.

[33] Strandness DE, Sumner DS (1975). Hemodynamics for surgeons.

[34] Shemesh D, Goldin I, Berelowitz D, Zaghal I, Zigelman C (2007). Blood flow volume changes in the maturing arteriovenous fistula for hemodialysis.. Ultrasound in Med Biol.

[35] Silva MB, Hobson RW, Pappas PJ, Jamil Z, Araki CT (1998). A strategy for increasing use of autogenous hemodialysis access procedures: impact of preoperative noninvasive evaluation.. J Vasc Surg.

[36] Mihmanli I, Besirli K, Kurugoglu S, Atakir K, Haider S (2001). Cephalic vein and hemodialysis fistula: surgeon's observation versus color Doppler ultrasonographic findings.. J Ultrasound Med.

[37] Reymond P, Bohraus Y, Perren F, Lazeyras F, Stergiopulos N Validation of a patient-specific one-dimensional model of the systemic arterial tree.. Am J Physiol Heart Circ Physiol.

[38] Bessems D, Giannopapa CG, Rutten MC, van de Vosse FN (2008). Experimental validation of a time-domain-based wave propagation model of blood flow in viscoelastic vessels.. J Biomech.

[39] Marchandise E, Willemet M, Lacroix V (2009). A numerical hemodynamic tool for predictive vascular surgery.. Med Eng Phys.

[40] Steele BN, Draney MT, Ku JP, Taylor CA (2003). Internet-based system for simulation-based medical planning for cardiovascular disease.. IEEE Trans Inf Technol Biomed.

[41] Planken RN, Leiner T, Nijenhuis RJ, Duijm LE, Cuypers PW (2008). Contrast-enhanced magnetic resonance angiography findings prior to hemodialysis vascular access creation: a prospective analysis.. J Vasc Access.

[42] Bode AS, Planken RN, Merkx MAG, van der Sande FM, Geerts L (2011). Feasibility of non contrast-enhanced magnetic resonance angiography for imaging upper extremity vasculature prior to vascular access creation.. Eur J Vasc Endovasc Surg.

[43] Roy-Chaudhury P, Spergel LM, Besarab A, Asif A, Ravani P (2007). Biology of arteriovenous fistula failure.. J Nephrol.

