# Supplementary material for: Patient-Specific Computational Modeling of Upper Extremity Arteriovenous Fistula Creation: Its Feasibility to Support Clinical Decision-Making
Source: PLoS One. 2012 Apr 4;7(4):e34491. doi: 10.1371/journal.pone.0034491 (PMC3319586; doi:10.1371/journal.pone.0034491)
Supplement: Appendix S1 — Monte Carlo simulations (see Robert et al.1 for details about Monte Carlo simulations) are used to determine the uncertainty in model predictions resulting from uncertainty in the input parameters assessed by measurements or from literature by applying certain assumptions. The uncertainties in the model parameters and their motivation are shown. (DOC) [file pone.0034491.s001.doc]

# Appendix 1

Monte Carlo simulations (see Robert et al.^1^ for details about Monte Carlo simulations) are used to determine the uncertainty in model predictions resulting from uncertainty in the input parameters assessed by measurements or from literature by applying certain assumptions. The uncertainties in the model parameters and their motivation are shown below.

| **Measurements** | **Uncertainty** | **Based on** |
| --- | --- | --- |
| Vessel diameters upper extremity | ± 10% | 2,3 |
| Brachial distensibility | ± 20% | 2 |
| Brachial pressure | ± 10% | 4 |
| Axillary, radial and ulnar artery flow | ± 15% | 5 |
| Mean aortic flow | ± 10% | 5 |

| **Assumptions** | **Uncertainty** | **Based on** |
| --- | --- | --- |
| Vessel lengths | ± 10% | Demographical data ([http://www.cbs.nl](http://www.cbs.nl/)) |
| Vessel diameter scaling factor | ± 20% | Retrospective patient cohort |
| Position of diameter measurements | ± 3 cm | Clinical experience |
| Wall thickness-radius ratio | ± 40% | 6 |
| Generic arterial Young’s modulus | ± 20% | 2 |
| Generic venous Young’s modulus | ± 20% | Assumed equal to arterial Young’s modulus |
| Anastomosis position | ± 2 cm | Clinical experience |
| Anastomosis angle | ± 20% | 7 |
| Time constants | ± 30% | 2 |
| Intravenous pressure | ± 50% | 8,9 |
| Generic mean windkessel flow | ± 15% | 10 |

# References

1. Robert CP, Casella GC. Monte Carlo statistical methods: Springer; 1999.

2. Leguy CA, Bosboom EM, Belloum AS, Hoeks AP, van de Vosse FN. Global sensitivity analysis of a wave propagation model for arm arteries. Med Eng Phys.

3. Li S, McDicken WN, Hoskins PR. Blood vessel diameter measurement by ultrasound. Physiol Meas 1993;14(3):291-297.

4. Eeftinck Schattenkerk DW, van Lieshout JJ, van den Meiracker AH, et al. Nexfin noninvasive continuous blood pressure validated against Riva-Rocci/Korotkoff. Am J Hypertens 2009;22(4):378-383.

5. Lotz J, Meier C, Leppert A, Galanski M. Cardiovascular flow measurement with phase-contrast MR imaging: basic facts and implementation. Radiographics 2002;22(3):651-671.

6. Kaiser DR, Mullen K, Bank AJ. Brachial artery elastic mechanics in patients with heart failure. Hypertension 2001;38(6):1440-1445.

7. Konner K. The anastomosis of the arteriovenous fistula--common errors and their avoidance. Nephrol Dial Transplant 2002;17(3):376-379.

8. Guyton AC, Hall JE. Textbook of medical physiology: WB Saunders; 2000.

9. Strandness DE, Jr., Sumner DS. Hemodynamics for surgeons: Grune and Stratton; 1975.

10. McArdle WD, Katch FI, V.L. K. Exercise physiology; energy, nutrition, and human performance: Williams and Wilkins; 1996.
